# Supplementary material for: The Systems Biology Research Tool: evolvable open-source software
Source: BMC Syst Biol. 2008 Jun 29;2:55. doi: 10.1186/1752-0509-2-55 (PMC2446383; doi:10.1186/1752-0509-2-55)
Supplement: Additional file 1 — SBRT Archive. An archive of the current version of the Systems Biology Research Tool. [file 1752-0509-2-55-S1.zip › sbrt-1.4.0/doc/developers_guide/api/sbrt/ext/R/stat/RCorrelator.Result.html]

RCorrelator.Result


|  |  |  |
| --- | --- | --- |
| **Overview** \| **Package** \| **Class** \| **Use** \| **Tree** \| **Deprecated** \| **Index** \| **Help** | |  |
| **PREV CLASS**   **NEXT CLASS** | **FRAMES**    **NO FRAMES**     **All Classes** |  |
| SUMMARY: NESTED \| FIELD \| CONSTR \| METHOD | DETAIL: FIELD \| CONSTR \| METHOD |  |


---


## sbrt.ext.R.stat Class RCorrelator.Result

```
java.lang.Object
  sbrt.ext.R.stat.RCorrelator.Result
```

**All Implemented Interfaces:**: CorrResult

**Enclosing class:**: RCorrelator<V>

---

``` public static final class RCorrelator.Result extends java.lang.Object implements CorrResult ```

This class is used to store the results of computing a correlation using
R.

**Author:**
:   This class was written and documented by Jeremiah Wright while in
    the Wagner lab.

---

| **Method Summary** | |
| --- | --- |
| `double` | `getCoeff()`             Returns the computed correlation coefficient. |
| `double` | `getP()`             Returns the corresponding P value. |
| `double` | `getStatistic()`             Returns the corresponding statistic. |
| `java.lang.String` | `toString()`             Returns a string representation of this result. |

**Methods inherited from class java.lang.Object**

`clone, equals, finalize, getClass, hashCode, notify, notifyAll, wait, wait, wait`

**Method Detail**

### getStatistic

```
public double getStatistic()
```

:   Returns the corresponding statistic.

    :   **Returns:**: the corresponding statistic.

---


### getCoeff

```
public double getCoeff()
```

:   Returns the computed correlation coefficient.

    :   **Specified by:**: `getCoeff` in interface `CorrResult`
    :   **Returns:**: the computed correlation coefficient.

---


### getP

```
public double getP()
```

:   Returns the corresponding P value.

    :   **Returns:**: the corresponding P value.

---


### toString

```
public java.lang.String toString()
```

:   Returns a string representation of this result.

    :   **Overrides:**: `toString` in class `java.lang.Object`
    :   **Returns:**: a string representation of this result.


---


|  |  |  |
| --- | --- | --- |
| **Overview** \| **Package** \| **Class** \| **Use** \| **Tree** \| **Deprecated** \| **Index** \| **Help** | |  |
| **PREV CLASS**   **NEXT CLASS** | **FRAMES**    **NO FRAMES**     **All Classes** |  |
| SUMMARY: NESTED \| FIELD \| CONSTR \| METHOD | DETAIL: FIELD \| CONSTR \| METHOD |  |


---
